# Shock index and shock index, pediatric age-adjusted as predictors of mortality in pediatric patients with trauma: A systematic review and meta-analysis

**DOI:** 10.1371/journal.pone.0307367

**Published:** 2024-07-18

**Authors:** Seo Hee Yoon, Sang-Jun Shin, Heeyeon Kim, Yun Ho Roh

**Affiliations:** 1 Department of Pediatrics, Severance Children’s Hospital, Yonsei University College of Medicine, Seoul, Korea; 2 Biostatistics Collaboration Unit, Department of Biomedical Systems Informatics, Yonsei University College of Medicine, Seoul, Korea; UT Health San Antonio: The University of Texas Health Science Center at San Antonio, UNITED STATES OF AMERICA

## Abstract

This study aimed to assess the predictive ability of the shock index (SI) and the shock index, pediatric age-adjusted (SIPA) for mortality among pediatric patients with trauma (aged ≤ 18 years). A systematic search used PubMed, Embase, and Cochrane Library databases to identify pertinent articles published from their inception to 13 February 2023. For each SI and SIPA, the pooled sensitivity, specificity, diagnostic odds ratio (DOR), and area under the summary receiver operating characteristic curve (AUC) with the corresponding 95% confidence intervals were calculated. We planned a priori meta-regression analyses to explore heterogeneity using the following covariates: country, clinical setting, type of center, data source, and cutoff value. Twelve studies were included based on the inclusion criteria. Among them, nine studies with 195,469 patients were included for the SIPA at the hospital, four studies with 4,970 patients were included for the pre-hospital SIPA, and seven studies with 606,445 patients were included to assess the ability of the SI in predicting mortality. The pooled sensitivity and specificity with 95% confidence interval for predicting mortality were as follows: 0.58 (0.44–0.70) and 0.72 (0.60–0.82), respectively, for the SIPA at the hospital; 0.61 (0.47–0.74) and 0.67 (0.61–0.73), respectively, for the pre-hospital SIPA; and 0.71 (0.59–0.81) and 0.45 (0.31–0.59), respectively for the SI. The DOR were 3.80, 3.28, and 2.06 for the SIPA at the hospital, pre-hospital SIPA, and SI, respectively. The AUC were 0.693, 0.689, and 0.618 for the SIPA at the hospital, pre-hospital SIPA, and SI, respectively. The SI and SIPA are simple predictive tools with sufficient accuracy that can be readily applied to pediatric patients with trauma, but SIPA and SI should be utilized cautiously due to their limited sensitivity and specificity, respectively.

## Introduction

Trauma is mostly caused by sudden injuries or accidents resulting in tissue injury [1]. It is a frequently overlooked primary cause contributing to mortality and disability in children and adolescents globally [2–4]. In the United States, injury-related causes accounted for > 60% of deaths among children and adolescents in 2016 [5, 6]. Trauma-related deaths are mostly preventable [7], thus timely triage to identify patients who require urgent care or referral to an advanced level of care is vital for improving clinical outcomes [8, 9].

The shock index (SI) is determined by dividing the heart rate (HR) by the systolic blood pressure (SBP). SI is a simple tool which can be readily employed during the initial evaluation and triage of patients with trauma, even in pre-hospital settings. The SI has been noted as a better diagnostic marker for detecting hemodynamic instability compared to conventional vital signs [10, 11]. Typically, the standard cutoff value for adults is 0.9 [12]; however, the vital signs of pediatric patients differ depending on age [13, 14]. Hence, the shock index, pediatric age-adjusted (SIPA) was developed. SIPA is calculated by dividing the maximum HR by the minimum SBP based on age [15, 16]. The SIPA has been reported to be better than the SI for predicting the need for surgery, use of mechanical ventilation, need for blood transfusion, admission to the intensive care unit, and mortality in pediatric patients with trauma [15, 17, 18]. Moreover, studies have reported the usefulness of the SI and SIPA in different clinical settings (e.g. warzone or civilian settings) and according to the recorded time (e.g. at the trauma scene [pre-hospital] or upon hospital arrival) [19–21]. Nevertheless, recent findings from observational studies involving pediatric patients have not been collectively analyzed and presented. Therefore, this study aims to offer an updated overview of the performance of the SI and SIPA in predicting mortality among pediatric patients with trauma.

## Methods

This study followed the Preferred Reporting Items for Systematic reviews and Meta-Analyses statement (PRISMA) [22] and was preregistered on International Prospective Register of Systematic Reviews (CRD42023380142). Ethical approval or consent to participate was not applicable because all data collection and analysis were based on already published studies.

### Search strategy, inclusion/exclusion criteria, and data extraction

Two researchers (SHY and HK) independently conducted a systematic search of the PubMed, Embase, and Cochrane Library databases for relevant articles published before 13 February 2023, without language restrictions. The detailed search strategies for each database are presented in S1 Table. Studies that investigated the ability of the SI or SIPA in predicting mortality among pediatric patients with trauma (aged ≤ 18 years) were eligible for inclusion. We only included studies that reported sufficient data for 2 × 2 tables. For each study selected, the collected data were as follows: name of the author(s), year of publication, country of publication, inclusion criteria (including participant characteristics), clinical settings, single or multicenter status, time (pre-hospital and hospital) and place of SI and SIPA measurement, cutoff values for the SI and SIPA, age-specific vital sign criteria (reference standards for HR and SBP) for SIPA, total number of patients, and true positives, false positives, true negatives, and false negatives of the SI and SIPA for predicting mortality. If datasets derived from separate study groups were present in a single study, each dataset was treated as a single study. However, for studies with more than two datasets derived from the same population, we selected only one dataset to avoid double-counting bias in our pooled analysis. If a study used different cutoff values in the same study population, we only selected one dataset that used one cutoff value of the SI or SIPA. When using the same national data registry, if study periods overlapped, we selected studies with a longer duration and that met the appropriate inclusion criteria better. Case reports, reviews, editorials, or animal or laboratory experiments were excluded.

The included observational studies were evaluated for validity using the Newcastle–Ottawa Scale (NOS)(scores range from 0–9) [23]. Each study was considered to be of low quality (a total score of ≤ 4), moderate quality (a total score between 5 and 6), or high quality (a total score of ≥ 7) [24]. Two authors (HK and SHY) independently appraised the quality of all the studies. Disagreements were discussed by the reviewers until an agreement was reached.

### Statistical analyses

For each SI and SIPA, we calculated the pooled sensitivity, specificity, and diagnostic odds ratio (DOR) with the corresponding 95% confidence intervals (CIs). The DOR represents the ratio of the odds of a positive test result among individuals with the disease to the odds of a positive test result among those without the disease [25, 26]. The range of the DOR is from 0 to infinity, and elevated values signify superior discriminative performance of the test [27]. When the DOR is greater than 1, the test result among subjects with disease is more likely to be positive than that in those without disease [25]. The area under the summary receiver operating characteristic curve (AUC) was also used to summarise the overall test accuracy. The AUC ranges from 0.5 to 1, and values approaching 0.5 suggest a non-discriminating test and higher values near 1.0 indicating a perfectly discriminating test [26, 28]. We used a forest plot to graphically summarise the information from each study [29]. The presence of heterogeneity in sensitivity and specificity was assessed using forest plots generated from the studies’ estimates and the Q-statistic (*p* < 0.1, significant). If significant heterogeneity was present, a priori planned subgroup analyses and meta-regression analyses were performed to investigate the sources of heterogeneity using the following covariates with 95% CIs: country (US *vs*. other countries), clinical setting (warzone/combat *vs*. civilian), type of center (single center *vs*. multicenter), data source (national data registry *vs*. medical records), and cutoff value (typical *vs*. new). According to previous studies [14, 15, 17–19, 30, 31], the following cutoff values (including very close cutoff values) were considered ‘typical’ cutoff values: 1.2 (ages 0–6 years), 1.0 (ages 7–12), and 0.9 (ages 13–18 years) for the SIPA and 0.9 for the SI. Other cutoff values were considered ‘new’ cutoff values for the meta-regression analysis. If a study included data from the US and other countries, and it was not conducted solely in the US, we classified that study as ’other countries’. Additionally, we performed the above pooled analysis according to the time of SI or SIPA measurement (at the trauma scene [pre-hospital] and at the hospital) to determine when it is more useful to measure the SI or SIPA. We employed funnel plots and conducted Egger’s tests to assess the possible publication bias. Statistical analyses were performed using R program, version 4.2.2 (R Foundation for Statistical Computing, Vienna, Austria). A p-value below 0.05 was deemed statistically significant.

## Results

**Fig 1:**
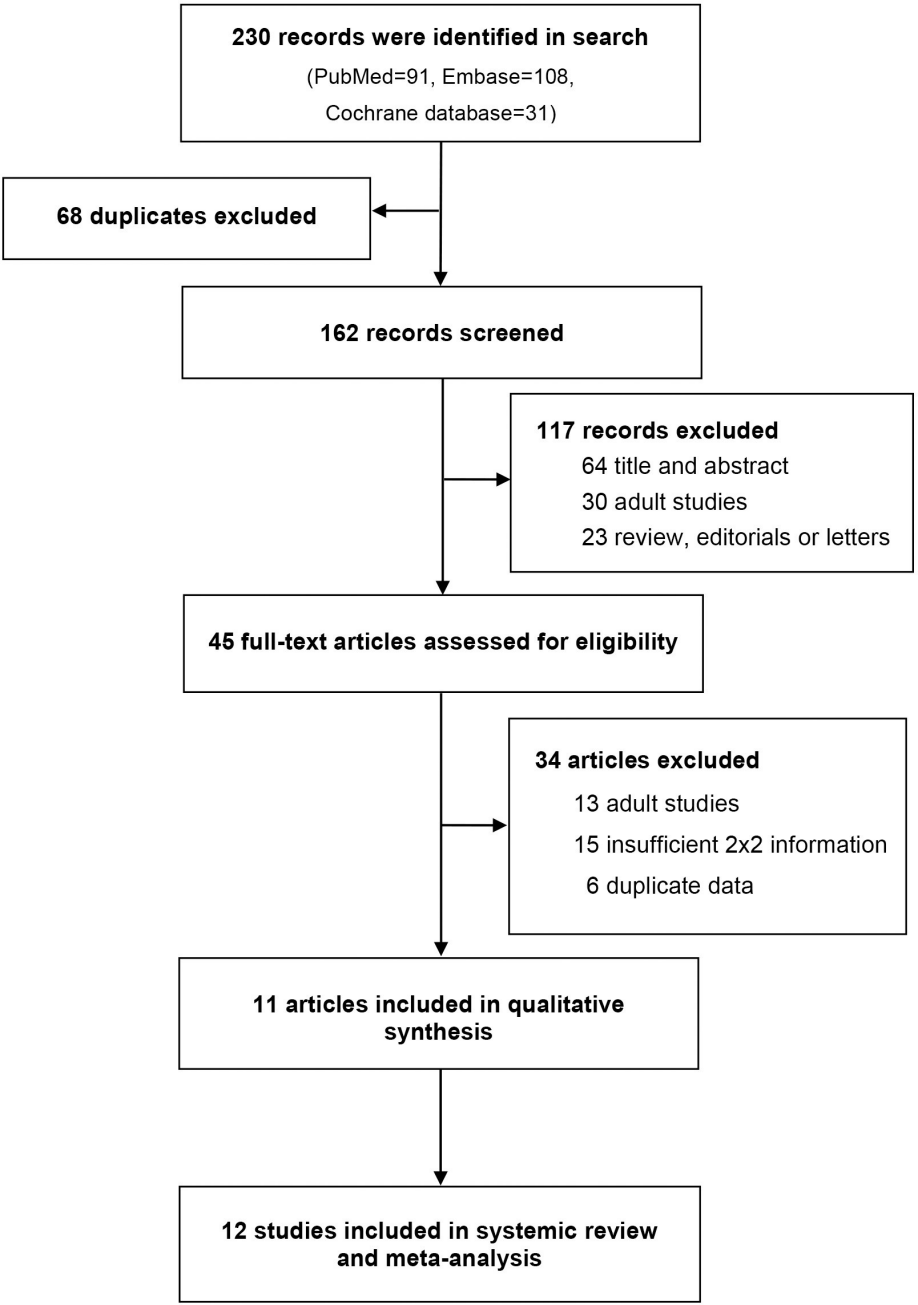
PRISMA flow chart presenting the search and selection process.

The electronic search of databases yielded 230 references (Fig 1). Subsequently, 68 duplicates were excluded. After screening the titles and abstracts, 45 studies underwent full-text evaluation, and 12 studies were included to our study. All studies were ranked as having moderate quality (S2 Table). The characteristics of the study are summarized in Table 1. The additional characteristics of the included studies are presented in S3 Table. The age-specific vital sign criteria for the SIPA in each included study are presented in S4 Table.

**Table 1:** Characteristics of the included studies.

**Pre-hospital SIPA (at the trauma scene)**									
**Study ID**	**Country**	**Clinical setting**	**Type of center**	**Study periods**	**Data source**	**Inclusion criteria**	**Cutoffs**		**Sample size**
**2019 Nordin [20]**	US	Civilian setting	Multicenter	2014 to 2016	TQIP-P	Pediatric blunt trauma patients (1–15 years) with an ISS ≥15; only patients who arrived in the ED 120 min or less after EMS arrival.	(1–6 y) 1.2; (7–12 y) 1.0; (>12 y) 0.9		2,917
**2021 Marenco [21]**	US and other countries	Warzone/Combat setting	Multicenter	2008 to 2015	DODTR	Patients aged 17 years and younger who were first treated in Role II or III combat surgical hospitals	(0–6 y) 1.2; (7–17 y) 0.9		669
**2022 Raythatha [32]**	Australia	Civilian setting	Single center	Jan 2015 to Aug 2020	Medical records/trauma registries	Trauma patients aged between 0 and 16 years old	(<1 y) 2.0; (1–3 y) 1.5; (4–6 y) 1.25; (7-12y) 1.0; (≥13y) > 0		307
**2022 Stevens [8]**	US	Civilian setting	Single center	Jan 2009 to Dec 2019	Medical records/trauma registries	Patients aged ≤18 years admitted to a Level I pediatric trauma center following trauma activation	(<1 y) 1.65; (1–6 y) 1.52; (7–12 y) 1.26; (>12 y) 1.6		1,072
**SIPA at the hospital**									
**Study id**	**Country**	**Clinical setting**	**Type of center**	**Study periods**	**Data source**	**Inclusion criteria**	**Cutoffs**		**Sample size**
**2015 Acker [15]**	US	Civilian setting	Multicenter	Jan 7 to Jun 13, 2014	Medical records/trauma registries	Children aged 4–16 years admitted for blunt trauma with an ISS >15	(4–6 y) 1.22; (7–12 y) 1.0; (13–16 y) 0.9		543
**2017 Linnaus [18]**	US	Civilian setting	Multicenter	Apr 2013 to Jan 2016	Medical records/trauma registries	Patients aged ≤18 years with blunt liver and/or spleen injury with an ISS >15	(4–6 y) 1.22; (7–12 y) 1.0; (13–16 y) 0.9		386
**2018 Vandewalle [19]**	US	Civilian setting	Single center	Jan 1, 2010, to Dec 31, 2015	Medical records/trauma registries	Patients sustaining blunt injuries with an ISS of ≥15	(4–6 y) 1.22; (7–12 y) 1.0; (13–16 y) 0.9		234
**2019 Traynor–a [14]**	South Africa	Civilian setting	Single center	2012 to 2017	Medical records/trauma registries	Injured patients aged 1–17 years at the time of admission	(1–6 y) 1.22; (7–12 y) 1.0; (13–17 y) 0.9		741
**2019 Traynor–b [14]**	US	Civilian setting	Single center	2012 to 2017	Medical records/trauma registries	Injured patients aged 1–17 years at the time of admission)	(1–6 y) 1.22; (7–12 y) 1.0; (13–17 y) 0.9		907
**2020 Marenco [31]**	US and other countries	Warzone/Combat setting	Multicenter	2008 to 2015	DODTR	Patients <18 years who received care for traumatic injuries	(0–6 y) 1.2; (7–17 y) 0.9		2,121
**2021 Austin [33]**	US	Civilian setting	Multicenter	2013 to 2016	NTDB	Children aged 1–14 years who sustained traumatic injuries	(1–6 y) 1.2; (7–12 y) 1.0; (13–14 y) 0.9		189,003
**2022 Raythatha [32]**	Australia	Civilian setting	Single center	Jan 2015 to Aug 2020	Medical records/trauma registries	Trauma patients aged between 0 and 16 years	(<1 y) 2.0; (1–3 y) 1.5; (4–6 y) 1.25; (7–12 y) 1.0; (≥13 y) > 0		462
**2022 Stevens [8]**	US	Civilian setting	Single center	Jan 2009 to Dec 2019	Medical records/trauma registries	Patients aged ≤18 years who were admitted to a Level I pediatric trauma center following trauma activation	(<1 y) 2.54; (1–6 y) 1.47; (7–12 y) 1.31; (>12 y) 1.24		1,072
**SI**									
**Study id**	**Country**	**Clinical setting**	**Type of center**	**Study periods**	**Data source**	**Inclusion criteria**	**Time**	**Cutoffs**	**Sample size**
**2015 Acker [15]**	US	Civilian setting	Multicenter	Jan 7 to Jun 13, 2014	Medical records/trauma registries	All pediatric blunt trauma patients (1–15 years) with an ISS ≥15; only patients who arrived in the ED 120 min or less after EMS arrival	At the hospital	0.9	543
**2017 Linnaus [18]**	US	Civilian setting	Multicenter	Apr 2013 to Jan 2016	Medical records/trauma registries	Patients aged ≤18 years with blunt liver and/or spleen injury with an ISS >15	At the hospital	0.9	386
**2019 Nordin [20]**	US	Civilian setting	Multicenter	2014 to 2016	TQIP-P	All pediatric blunt trauma patients (1–15 years) with an ISS ≥15; only patients who arrived in the ED 120 min or less after EMS arrival	At the trauma scene	0.9	2,917
**2019 Traynor–a [14]**	South Africa	Civilian setting	Single center	2012 to 2017	Medical records/trauma registries	All injured patients aged 1–17 years at the time of admission	At the hospital	0.9	741
**2019 Traynor–b [14]**	US	Civilian setting	Single center	2012 to 2017	Medical records/trauma registries	All injured patients aged 1–17 years at the time of admission	At the hospital	0.9	907
**2020 Marenco [31]**	US and other countries	Warzone/Combat setting	Multicenter	2008 to 2015	DODTR	All patients aged <18 years who received care for traumatic injuries	At the hospital	0.8	2,121
**2022 Georgette [34]**	US	Civilian setting	Multicenter	2010 to 2018	TQP-PUF/NTDB	Patients aged 1–16 years with blunt or penetrating (excluding burn-dominant) injuries who were admitted to the hospital	At the hospital	0.9	598,830

DODTR = Department of Defense Trauma Registry, EMS = Emergency Medical Services, ED = Emergency Department, ISS = Injury Severity Score, NTDB = National Trauma Data Bank, SI = shock index, SIPA = shock index, pediatric age-adjusted, TQIP-P = Pediatric Trauma Quality Improvement Program database, TQP-PUF = Trauma Quality Program Participant Use File, US = United States.

### Pre-hospital SIPA to predict mortality

**Fig 2:**
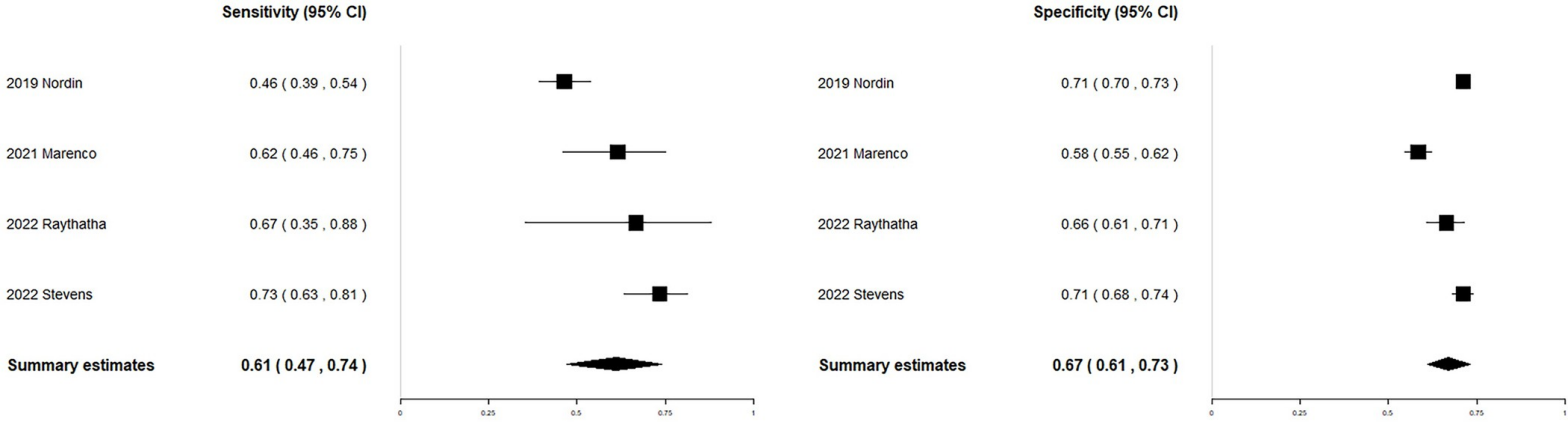
Coupled forest plots of the sensitivity and specificity of the SIPA (at the trauma scene or pre-hospital) for predicting mortality in pediatric patients with trauma. CI confidence interval, SIPA shock index, pediatric age-adjusted.

Four studies [8, 20, 21, 32] including 4,965 patients evaluated the performance of pre-hospital SIPA in predicting mortality (Table 1). The sensitivities (0.46–0.73) and specificities (0.58–0.71) of the included studies are visually presented in a forest plot (Fig 2). The pooled sensitivity and specificity of the pre-hospital SIPA for predicting mortality were 0.61 (95% CI, 0.47–0.74) and 0.67 (95% CI, 0.61–0.73), respectively. The DOR was 3.28 (95% CI, 1.76–6.12) (S1 Fig). The AUC was 0.689 (S2 Fig). There was substantial heterogeneity in sensitivity and specificity (Q-statistic, < 0.001). In subgroup analysis (S5 Table), the pooled sensitivities were higher in the following studies: studies conducted at a single center, studies using medical records/trauma registries (non-national), and studies using new cutoff values. The pooled specificities were higher in studies conducted in the US and in a civilian setting. The pooled sensitivities and specificities were similar across other subgroups. Through meta-regression analysis, among the covariates, the type of center, data source, and cutoff value significantly contributed to the heterogeneity in sensitivity, whereas the country and clinical setting (e.g., warzone or civilian) significantly affected in the heterogeneity in specificity (S5 Table). There was no evidence of publication bias observed, as indicated by the analysis of funnel plots and Egger’s test (*p* = 0.8066) (S3 Fig).

### SIPA at the hospital to predict mortality

**Fig 3:**
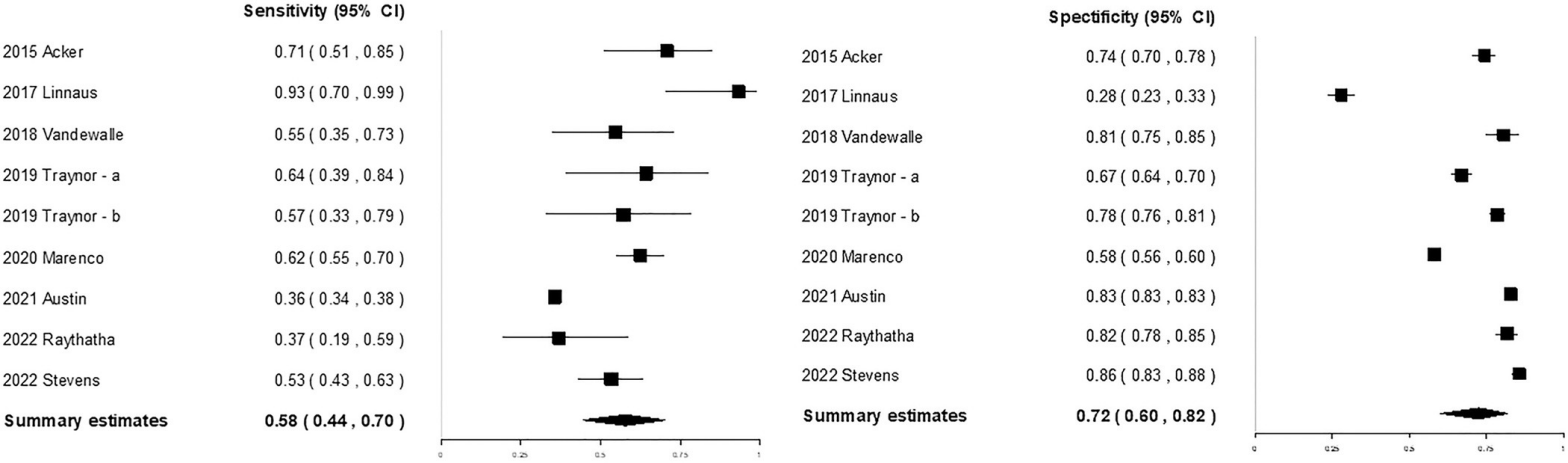
Coupled forest plots of the sensitivity and specificity of the SIPA (at the hospital) for predicting mortality in pediatric patients with trauma. CI confidence interval, SIPA shock index, pediatric age-adjusted.

Nine studies [8, 14, 15, 18, 19, 31–33] including 195,469 patients evaluated the performance of the SIPA at the hospital for predicting mortality (Table 1). The sensitivities (0.36–0.93) and specificities (0.28–0.86) of the included studies widely varied (Fig 3). The pooled sensitivity and specificity of the SIPA at the hospital in predicting mortality were 0.58 (95% CI, 0.44–0.70) and 0.72 (95% CI, 0.60–0.82), respectively. The DOR was 3.80 (95% CI, 2.66–5.42) (S4 Fig). The AUC was 0.693 (S5 Fig). There was substantial heterogeneity in sensitivity and specificity (Q-statistic, < 0.001). In subgroup analysis (S6 Table), higher pooled sensitivities were observed in the following studies: studies conducted in the US, studies conducted in the multicenter, studies using medical records/trauma registries (non-national), and studies using typical cutoff values. Additionally, higher pooled specificities were observed in the following studies: studies conducted in the US, studies conducted in a civilian setting, studies conducted at a single center, and studies using new cutoff values. The pooled sensitivities and specificities were similar across other subgroups. Meta-regression analysis did not identify any specific covariates contributing to the heterogeneity in sensitivity or specificity (S6 Table). There was no evidence of publication bias observed, as indicated by the analysis of funnel plots and Egger’s test (*p* = 0.256) (S6 Fig).

### SI to predict mortality

**Fig 4:**
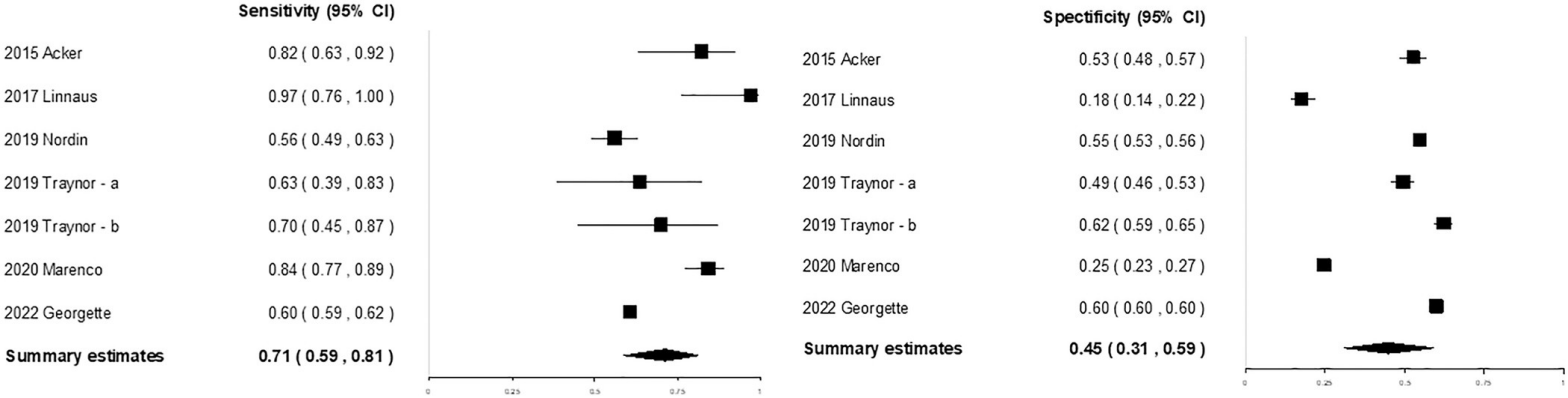
Coupled forest plots of the sensitivity and specificity of the SI for predicting mortality in pediatric patients with trauma. CI confidence interval, SI shock index.

Seven studies [14, 15, 18, 20, 31, 34] including 606,445 patients evaluated the performance of the SI for predicting mortality (Table 1). Among them, only one study evaluated pre-hospital SI; thus, data were not separately pooled according to the time of SI measurement. Instead, subgroup analysis and meta-regression analyses were performed with the time of SI measurement as a covariate. The sensitivities (0.60–0.97) and specificities (0.18–0.62) of the included studies are visually displayed in a forest plot (Fig 4). The pooled sensitivity and specificity of the SI for predicting mortality were 0.71 (95% CI, 0.59–0.81) and 0.45 (95% CI, 0.31–0.59), respectively. The DOR was 2.06 (95% CI, 1.60–2.65) (S7 Fig). The AUC was 0.618 (S8 Fig). There was substantial heterogeneity in sensitivity and specificity (Q-statistic, < 0.001). In subgroup analysis (S7 Table), the pooled sensitivities were higher in the following studies: studies conducted in a warzone/combat setting, studies conducted in the multicenter, studies using medical records/trauma registries (non-national), studies using new cutoff values and studies conducted at the hospital. The pooled sensitivities were similar between the studies conducted in the US and other countries. However, the pooled specificities remained consistently low, not exceeding 0.6 in any of the subgroups. This suggests that the comparison lacks clinically significant. No specific covariates were identified as contributors to the heterogeneity in sensitivity or specificity through meta-regression analysis (S7 Table). There was no evidence of publication bias observed, as indicated by the analysis of funnel plots and Egger’s test (*p* = 0.223) (S9 Fig).

## Discussion

In this study, we investigated the predictive ability of the SI and SIPA in assessing mortality among pediatric patients with trauma. Although the sensitivities and specificities of the SI and SIPA varied substantially across studies, SIPA exhibited low pooled sensitivities and moderate pooled specificities, whereas the SI demonstrated moderate pooled sensitivities and low pooled specificities for predicting mortality. Therefore, our results indicate that a positive SI can serve as a useful tool in identifying patients at high risk of mortality. Additionally, our findings suggest that a normal SIPA may be a useful tool in identifying patients at low risk of mortality. In addition, the AUC values ranging from 0.618 to 0.693 suggest that the SI and SIPA have sufficient discriminatory ability [26].

Recently, Vang et al. [35] reported the pooled risk of in-hospital mortality was higher with an SI of ≥1 than with an SI of <1 (pooled risk ratio: 4.15) in adult patients with trauma (age ≥16 years). Further, Carsetti et al. [36] found that an SI showed an overall sensitivity and specificity of 0.358 and 0.742, respectively, with an AUC value of 0.553 in predicting mortality in adult patients with trauma (age ≥14 years). These results showed lower sensitivity and accuracy, but higher specificity compared to our findings regarding the predictive performance of SI in pediatric patients with trauma (age ≤18 years). Furthermore, in our current study, the SIPA demonstrated higher predictive accuracy compared to the SI, as reported by Carsetti et al. [36]. The authors [36] thus recommended considering alternative scores to the SI due to its limited capacity for predicting mortality in adult patients with trauma. Interestingly, age was suggested as one of the important factors affecting the prediction capacity of several scores for mortality (e.g. older adults are at increased risk of mortality despite having less severe injuries) [36, 37]. Bruijns et al. [37] reported that conventional vital signs combined with age (e.g. SI multiplied by age, HR / (220 − age), and SBP / age) were better at predicting mortality in patients with blunt injurie than HR, SBP, or age by itself.

When compared with SI, both pre-hospital SIPA and SIPA at the hospital demonstrated lower pooled sensitivities but higher pooled specificities. Due to its higher specificity, a positive SIPA can better rule in patients at risk of mortality, as it gives fewer false-positive results compared to a positive SI. However, the lower pooled sensitivity of the SIPA suggests that due to more false-negative results, a normal SIPA may miss some patients at risk of death that the SI would have identified [13, 17]. On the contrary, thanks to its higher sensitivity, a positive SI can better identify patients at high risk of mortality compared to SIPA. However, its lower specificity may result in some low-risk patients being falsely diagnosed as high-risk due to its high false positive results. Additionally, a normal SI can effectively rule out patients at high risk of mortality with fewer false-negative results. Consequently, using SI as a screening tool to identify patients at high risk of mortality, followed by utilizing SIPA as a confirmation tool, may be beneficial.

When comparing war/combat and civilian settings, the pooled specificities of SIPA (pre-hospital and at the hospital) exhibited similar sensitivity between warzone/combat and civilian settings but demonstrated higher pooled specificity in the civilian setting compared to the warzone/combat setting. Conversely, SI studies demonstrated higher pooled sensitivity in the warzone/combat setting than in the civilian setting. Pooled specificity was higher in the civilian setting, although it remained below 0.5. These findings suggest that a normal SIPA can effectively identify patients at low risk of mortality, while a positive SIPA can be more useful in confirming patients at risk of death in a civilian setting. Additionally, a positive SI can be more useful in identifying patients at risk of death, and a normal SI can effectively rule out patients at high risk of mortality in a warzone setting. Within the warzone/combat setting, the data included patients with multiple injuries, mostly penetrating or blast injuries. Different injury patterns and the accessibility of emergency medical services/healthcare facilities between the warzone/combat and civilian settings may have influenced these results.

Cutoff of SIPA is calculated from the maximum normal heart rate and the minimum normal SBP. When comparing the typical and new cutoff values of the SIPA, studies using the SIPA with the new cutoff values demonstrated a high pooled specificity (0.838) when assessed at the hospital in our results. Therefore, it appears more advantageous to employ the SIPA with the new cutoff values, especially in hospital setting. Considering these results, it is necessary to re-establish and apply new normal pediatric vital signs [8, 32] rather than using the previously known normal vital signs based on age [14, 15, 18, 19, 31, 33]. As there are not many studies using the new cutoff value yet, additional studies and validation are warranted.

Regarding the time of measurement, while SIPA showed similar pooled sensitivity in the pre-hospital and hospital settings, and the pooled specificity was slightly higher in the hospital setting. Regarding SI, only one included study [20] evaluated SI’s predictive performance in the pre-hospital setting, making accurate comparisons challenging. However, results from this study showed lower sensitivity but slightly higher specificity compared to the pooled results from the hospital settings. The disparities in outcome results between pre-hospital and hospital settings suggest the importance of considering measurement timing and indicate the need for further research.

Recently, several prognostic tools designed to predict mortality in pediatric patients with trauma have been proposed. Vandewalle et al. analysed SIPA trends in pediatric patients aged 4 to 16 years with severe (Injury Severity Score; ISS ≥ 15) and moderate (ISS 10–14) blunt injuries. They found that patients with severe blunt injuries initially presenting with a normal SIPA, but later developing a positive SIPA within 24 hours, had a higher mortality rate compared to those with consistently normal SIPA within the first 48 hours of admission [19]. Similarly, patients with moderate blunt injuries, initially presenting with a normal SIPA upon arrival, but subsequently developing a positive SIPA within 24 hours, were associated with an increased length of stay and the need for transfusion within the first 24 hours of arrival [38]. Moreover, Marenco et al. [21] reviewed the Department of Defense Trauma Registry for pediatric patients with warzone trauma (age ≤ 17 years) and found that patients with a positive SIPA at both pre-hospital and arrival to initial care showed increased incidence of intensive care unit (ICU) admission, mechanical ventilation requirement, and mortality. Nordin et al. [20] compared SI and SIPA values at the trauma scene and in the ED for blunt trauma patients aged 1 to 15 years with ISS > 15. Persistently abnormal SIPA was found to have stronger associations with overall length of stay, ICU admission, ventilator use, and mortality than persistently abnormal SI.

Furthermore, the reverse shock index multiplied by the Glasgow Coma Scale score (rSIG) has shown promise in adult trauma trauma [39, 40]. Lammers et al. [41] discovered that rSIG outperformed SIPA as an independent predictor for early mortality in pediatric patients with warzone injuries, with odds ratios of 4.054 (p = 0.01) and 2.742 (p < 0.01), respectively. Lammers et al. [42] also found that a positive rSIG exhibited higher sensitivity (91.7%) and specificity (79.0%) for mortality prediction in civilian pediatric patients compared to a positive SIPA, which had a sensitivity of 55.5% and specificity of 70.2%. Reppucci et al. [43] examined the performance of rSIG in predicting early interventions in civilian pediatric trauma patients and its role in trauma triage. They found that rSIG surpassed traditional SI and SIPA in identifying traumatically injured children at risk for early interventions. Additionally, Reppucci et al. [44] compared two novel pediatric trauma scoring tools, SIPAB+ (elevated SIPA with GCS ≤8) and rSIG, to determine which more accurately identifies children requiring pediatric trauma team activation. SIPAB+ upon ED arrival exhibited high specificity (>98%) but poor sensitivity (<30%), while abnormal rSIG upon ED arrival balanced both sensitivity (>60%) and specificity (>60%). Furthermore, Reppucci et al. [45] investigated the use of rSIG in the prehospital setting for identifying severely injured children needing specialized trauma care. They found that abnormal prehospital rSIG was associated with need for interventions such as intubation, blood transfusion, intracranial pressure monitoring/drain, and admissions to the intensive care unit, suggesting its potential role in early identification and triage of severely injured children by emergency medical service providers.

Recently, Smida et al. [46] investigated the predictive performance of the reverse shock index times the motor component of the Glasgow Coma Scale (rSIM) compared to rSIG in pediatric trauma. Using data from the 2017–2020 National Trauma Data Bank, they included patients aged 1–16 years with documented prehospital and ED vital signs and total GCS. Their primary outcome was in-hospital mortality, while secondary outcomes included blood product administration, hemorrhage control intervention, and ICU admission from the ED. After analyzing 77,996 patients, they found that rSIM and rSIG performed similarly in predicting mortality across young age groups (1–2 and 3–5 years), with statistically superior performance of rSIG in older age groups (6–12 and 13–16 years). Both tools showed comparable performance in predicting secondary outcomes. Considering its simplicity, the authors suggested that rSIM may serve as a useful tool for pediatric trauma triage. Those results suggest that considering neurological status is crucial in the initial assessment of patients.

Our main strength lay in incorporating a substantial sample size and assessing the performance of both the SI and SIPA in pediatric patients with trauma, which would yield more accurate results compared to previous studies. In addition, we encompassed studies with different clinical settings, data sources, and cutoffs. However, this study has several limitations. Firstly, it’s noteworthy that all the studies included in this analysis were observational, inherently carrying an unavoidable risk of bias, although they were assessed as being of moderate quality using the NOS. Second, the lack of randomisation in our included studies led to significant heterogeneity; nevertheless, we performed subgroup and meta-regression analyses to explore the sources of heterogeneity. Third, all included studies excluded individuals without recorded HR or SBP data upon arrival at the hospital. If the studies included casualties who died pre-hospital or were pronounced dead on arrival, this may have affected our findings. Finally, due to limited information, we were unable to investigate the pooled results of the followings: the usefulness of SIPA trends, a comparison of SIPA with alternative scoring systems, and the assessment of SIPA based on the severity of trauma or injury characteristics for predicting mortality. Georgette et al. [34] reported that the SIPA had a remarkably low positive predictive value for mortality in pediatric spinal injury. The authors presumed that neurogenic shock from spinal cord injury might result in bradycardia, and consequently a ‘normal’ SIPA notwithstanding critical injury. Therefore, the application of the SI or SIPA to pediatric patients with spinal cord injury needs to require cautions and further studies.

## Conclusion

The SI and SIPA are simple predictive tools with sufficient accuracy, enabling their application even in resource-poor environments. A normal SIPA may be particularly useful for identifying pediatric patients with trauma at low risk of mortality, while a positive SIPA can confirm those at high risk compared to SI. Conversely, a positive SI may be particularly useful in identifying pediatric patients with trauma at high risk of mortality, whereas a normal SI can rule out those at high risk compared to SIPA. However, the limited sensitivity of SIPA and the limited specificity of SI restrict their use as the sole screening parameters. Instead, it is also worth considering using SI as a screening tool and SIPA as a confirming tool, together. Further studies are necessary to assess the utility and validate new tools such as rSIG, rSIM, and trends in SIPA across diverse clinical settings, along with investigating new cutoff values for SIPA.

## Supporting information

S1 Checklist PRISMA checklist. (DOC)

S1 Table Search strategy. (DOCX)

S2 Table Study quality assessment using the Newcastle-Ottawa Scale. (DOCX)

S3 Table Additional characteristics of each included study. (DOCX)

S4 Table Normal pediatric vital signs based on age with calculated SIPA cutoff values. (DOCX)

S5 Table Subgroup analysis and meta-regression analysis (pre-hospital SIPA). (DOCX)

S6 Table Subgroup analysis and meta-regression analysis (SIPA at the hospital). (DOCX)

S7 Table Subgroup analysis and meta-regression analysis (SI). (DOCX)

S1 Fig Forest plots of the diagnostic odds ratio of the SIPA (at the trauma scene or pre-hospital) in predicting mortality among pediatric patients with trauma. CI = confidence interval, SIPA = shock index, pediatric age-adjusted.
(TIF)

S2 Fig Summary receiver operating characteristic (SROC) curve illustrating the predictive performance of the SIPA (at the trauma scene or pre-hospital) for mortality in pediatric patients with trauma. The area under the curve of the SROC was 0.689. SIPA = shock index, pediatric age-adjusted.
(TIF)

S3 Fig Funnel plot of the included studies (at the trauma scene or pre-hospital). There was no significant publication bias observed by Egger’s test (p = 0.8066). SIPA = shock index, pediatric age-adjusted.
(TIF)

S4 Fig Forest plots of the diagnostic odds ratio of the SIPA (at the hospital) in predicting mortality among pediatric patients with trauma. CI = confidence interval, SIPA = shock index, pediatric age-adjusted.
(TIF)

S5 Fig Summary receiver operating characteristic (SROC) curve illustrating the predictive performance of the SIPA (at the hospital) for mortality in pediatric patients with trauma. The area under the curve of the SROC was 0.693. SIPA = shock index, pediatric age-adjusted.
(TIF)

S6 Fig Funnel plot of the included studies (SIPA at the hospital). There was no significant publication bias observed by Egger’s test (p = 0.256). SIPA = shock index, pediatric age-adjusted.
(TIF)

S7 Fig Forest plots of the diagnostic odds ratio of the SI in predicting mortality among pediatric patients with trauma. CI = confidence interval, SI = shock index.
(TIF)

S8 Fig Summary receiver operating characteristic (SROC) curve illustrating the predictive performance of the SI for mortality in pediatric patients with trauma. The area under the curve of the SROC was 0.618. SI = shock index.
(TIF)

S9 Fig Funnel plot of the included studies (SI). There was no significant publication bias observed by Egger’s test (p = 0.223). SI = shock index.
(TIF)
