# Supplementary material for: Shock index and shock index, pediatric age-adjusted as predictors of mortality in pediatric patients with trauma: A systematic review and meta-analysis
Source: PLoS One. 2024 Jul 18;19(7):e0307367. doi: 10.1371/journal.pone.0307367 (PMC11257222; doi:10.1371/journal.pone.0307367)
Supplement: S1 Table — (DOCX) [file pone.0307367.s002.docx]

**S1 Table. Search strategy**

**<PubMed>**

| Search number | Query | Search Details |
| --- | --- | --- |
| 5 | (((("Wounds and Injuries"[Mesh]) OR ((("Trauma"[tiab]) OR ("Injury"[tiab])) OR ("Injured"[tiab]))) AND (((((("Child"[Mesh]) OR ("Adolescent"[Mesh])) OR ("Infant, Newborn"[Mesh])) OR ("Infant"[Mesh])) OR ("Child, Preschool"[Mesh])) OR (((((((("pediatric"[tiab]) OR ("paediatric"[tiab])) OR ("neonat*"[tiab])) OR ("newborn"[tiab])) OR ("infant"[tiab])) OR ("child"[tiab])) OR ("preschool"[tiab])) OR ("adolescent"[tiab])))) AND ((("Shock index"[tiab]) OR ("shock index pediatric age-adjusted"[tiab])) OR ("SIPA"[tiab]))) AND ((("Death"[Mesh]) OR "Mortality"[Mesh]) OR (("Mortality"[tiab]) OR "Death"[tiab])) | ("Wounds and Injuries"[MeSH Terms] OR ("Trauma"[Title/Abstract] OR "Injury"[Title/Abstract] OR "Injured"[Title/Abstract])) AND ("Child"[MeSH Terms] OR "Adolescent"[MeSH Terms] OR "infant, newborn"[MeSH Terms] OR "Infant"[MeSH Terms] OR "child, preschool"[MeSH Terms] OR ("pediatric"[Title/Abstract] OR "paediatric"[Title/Abstract] OR "neonat*"[Title/Abstract] OR "newborn"[Title/Abstract] OR "Infant"[Title/Abstract] OR "Child"[Title/Abstract] OR "preschool"[Title/Abstract] OR "Adolescent"[Title/Abstract])) AND ("Shock index"[Title/Abstract] OR "shock index pediatric age-adjusted"[Title/Abstract] OR "SIPA"[Title/Abstract]) AND ("Death"[MeSH Terms] OR "Mortality"[MeSH Terms] OR ("Mortality"[Title/Abstract] OR "Death"[Title/Abstract])) |
| 4 | (("Death"[Mesh]) OR "Mortality"[Mesh]) OR (("Mortality"[tiab]) OR "Death"[tiab]) | "Death"[MeSH Terms] OR "Mortality"[MeSH Terms] OR "Mortality"[Title/Abstract] OR "Death"[Title/Abstract] |
| 3 | (("Shock index"[tiab]) OR ("shock index pediatric age-adjusted"[tiab])) OR ("SIPA"[tiab]) | "Shock index"[Title/Abstract] OR "shock index pediatric age-adjusted"[Title/Abstract] OR "SIPA"[Title/Abstract] |
| 2 | ((((("Child"[Mesh]) OR ("Adolescent"[Mesh])) OR ("Infant, Newborn"[Mesh])) OR ("Infant"[Mesh])) OR ("Child, Preschool"[Mesh])) OR (((((((("pediatric"[tiab]) OR ("paediatric"[tiab])) OR ("neonat*"[tiab])) OR ("newborn"[tiab])) OR ("infant"[tiab])) OR ("child"[tiab])) OR ("preschool"[tiab])) OR ("adolescent"[tiab])) | "Child"[MeSH Terms] OR "Adolescent"[MeSH Terms] OR "infant, newborn"[MeSH Terms] OR "Infant"[MeSH Terms] OR "child, preschool"[MeSH Terms] OR "pediatric"[Title/Abstract] OR "paediatric"[Title/Abstract] OR "neonat*"[Title/Abstract] OR "newborn"[Title/Abstract] OR "Infant"[Title/Abstract] OR "Child"[Title/Abstract] OR "preschool"[Title/Abstract] OR "Adolescent"[Title/Abstract] |
| 1 | ("Wounds and Injuries"[Mesh]) OR ((("Trauma"[tiab]) OR ("Injury"[tiab])) OR ("Injured"[tiab])) | "Wounds and Injuries"[MeSH Terms] OR "Trauma"[Title/Abstract] OR "Injury"[Title/Abstract] OR "Injured"[Title/Abstract] |

**<Embase>**

| No. | Query |
| --- | --- |
| #11 | (('wounds and injuries'/exp OR 'wounds and injuries') OR ('trauma':ti,ab OR 'injury':ti,ab)) AND ('shock index':ti,ab OR 'shock index pediatric age-adjusted':ti,ab OR 'sipa':ti,ab) AND (('child'/exp OR 'adolescent'/exp OR 'newborn'/exp OR 'infant'/exp OR 'preschool child'/exp OR 'neonate'/exp) OR ('pediatric':ti,ab OR 'paediatric':ti,ab OR 'neonat*':ti,ab OR 'newborn':ti,ab OR 'infant':ti,ab OR 'child':ti,ab OR 'preschool':ti,ab OR 'adolescent':ti,ab)) AND (('death'/exp OR 'mortality'/exp) OR ('mortality':ti,ab OR 'death':ti,ab)) |
| #10 | ('death'/exp OR 'mortality'/exp) OR ('mortality':ti,ab OR 'death':ti,ab) |
| #9 | 'mortality':ti,ab OR 'death':ti,ab |
| #8 | 'death'/exp OR 'mortality'/exp |
| #7 | ('child'/exp OR 'adolescent'/exp OR 'newborn'/exp OR 'infant'/exp OR 'preschool child'/exp OR 'neonate'/exp) OR ('pediatric':ti,ab OR 'paediatric':ti,ab OR 'neonat*':ti,ab OR 'newborn':ti,ab OR 'infant':ti,ab OR 'child':ti,ab OR 'preschool':ti,ab OR 'adolescent':ti,ab) |
| #6 | 'pediatric':ti,ab OR 'paediatric':ti,ab OR 'neonat*':ti,ab OR 'newborn':ti,ab OR 'infant':ti,ab OR 'child':ti,ab OR 'preschool':ti,ab OR 'adolescent':ti,ab |
| #5 | 'child'/exp OR 'adolescent'/exp OR 'newborn'/exp OR 'infant'/exp OR 'preschool child'/exp OR 'neonate'/exp |
| #4 | 'shock index':ti,ab OR 'shock index pediatric age-adjusted':ti,ab OR 'sipa':ti,ab |
| #3 | ('wounds and injuries'/exp OR 'wounds and injuries') OR ('trauma':ti,ab OR 'injury':ti,ab) |
| #2 | 'trauma':ti,ab OR 'injury':ti,ab |
| #1 | 'wounds and injuries'/exp OR 'wounds and injuries' |

**< Cochrane Library >**

| No. | Search |
| --- | --- |
| #1 | MeSH descriptor: [Wounds and Injuries] explode all trees |
| #2 | (Trauma):ti,ab,kw |
| #3 | (Injury):ti,ab,kw |
| #4 | (Injured):ti,ab,kw |
| #5 | #1 OR #2 OR #3 OR #4 |
| #6 | MeSH descriptor: [Child] explode all trees |
| #7 | MeSH descriptor: [Adolescent] explode all trees |
| #8 | MeSH descriptor: [Infant, Newborn] explode all trees |
| #9 | MeSH descriptor: [Infant] explode all trees |
| #10 | MeSH descriptor: [Child, Preschool] explode all trees |
| #11 | (pediatric):ti,ab,kw |
| #12 | (paediatric):ti,ab,kw |
| #13 | (newborn):ti,ab,kw |
| #14 | (neonate):ti,ab,kw |
| #15 | (infant):ti,ab,kw |
| #16 | (child):ti,ab,kw |
| #17 | (preschool):ti,ab,kw |
| #18 | (adolescent):ti,ab,kw |
| #19 | #6 OR #7 OR #8 OR #9 OR #10 OR #11 OR #12 OR #13 OR #14 OR #15 OR #16 OR #17 OR #18 |
| #20 | MeSH descriptor: [Death] explode all trees |
| #21 | MeSH descriptor: [Mortality] explode all trees |
| #22 | (Mortality):ti,ab,kw |
| #23 | (Death):ti,ab,kw |
| #24 | #20 OR #21 OR #22 OR #23 |
| #25 | (Shock index):ti,ab,kw |
| #26 | (shock index pediatric age-adjusted):ti,ab,kw |
| #27 | (SIPA):ti,ab,kw |
| #28 | #25 OR #26 OR #27 |
| #29 | #5 AND #19 AND #24 AND #28 |
