# Supplementary material for: Shock index and shock index, pediatric age-adjusted as predictors of mortality in pediatric patients with trauma: A systematic review and meta-analysis
Source: PLoS One. 2024 Jul 18;19(7):e0307367. doi: 10.1371/journal.pone.0307367 (PMC11257222; doi:10.1371/journal.pone.0307367)
Supplement: S2 Table — (DOCX) [file pone.0307367.s003.docx]

**S2 Table. Study quality assessment using the Newcastle-Ottawa Scale**

| **Author** | **Year** | **Selection** | | | | **Comparability** | **Exposure** | | | **Total score** |
| --- | --- | --- | --- | --- | --- | --- | --- | --- | --- | --- |
|  |  | **Adequate definition of patient cases** | **Representativeness of patient cases** | **Selection of controls** | **Definition of controls** | **Control for important or additional factors** | **Ascertainment of exposure** | **Same method of ascertainment for participants** | **Nonresponse rate** |  |
| **2015 Acker** | **2015** | **⋆** | **-** | **-** | **⋆** | **⋆** | **⋆** | **⋆** | **-** | **5** |
| **2017 Linnaus** | **2017** | **⋆** | **-** | **-** | **⋆** | **⋆** | **⋆** | **⋆** | **-** | **5** |
| **2018 Vandewalle** | **2018** | **⋆** | **⋆** | **-** | **⋆** | **⋆** | **⋆** | **⋆** | **-** | **6** |
| **2019 Nordin** | **2019** | **⋆** | **⋆** | **-** | **⋆** | **⋆** | **⋆** | **⋆** | **-** | **6** |
| **2019 Traynor - a** | **2019** | **⋆** | **⋆** | **-** | **⋆** | **⋆** | **⋆** | **⋆** | **-** | **6** |
| **2019 Traynor - b** | **2019** | **⋆** | **⋆** | **-** | **⋆** | **⋆** | **⋆** | **⋆** | **-** | **6** |
| **2020 Marenco** | **2020** | **⋆** | **⋆** | **-** | **⋆** | **⋆** | **⋆** | **⋆** | **-** | **6** |
| **2021 Austin** | **2021** | **⋆** | **⋆** | **-** | **⋆** | **⋆** | **⋆** | **⋆** | **-** | **6** |
| **2021 Marenco** | **2021** | **⋆** | **⋆** | **-** | **⋆** | **⋆** | **⋆** | **⋆** | **-** | **6** |
| **2022 Georgette** | **2022** | **⋆** | **-** | **-** | **⋆** | **⋆** | **⋆** | **⋆** | **-** | **5** |
| **2022 Raythatha** | **2022** | **⋆** | **⋆** | **-** | **⋆** | **⋆** | **⋆** | **⋆** | **-** | **6** |
| **2022 Stevens** | **2022** | **⋆** | **⋆** | **-** | **⋆** | **⋆** | **⋆** | **⋆** | **-** | **6** |

*Each star indicates a point for each component. Any study can obtain a maximum of four, two and three stars for each component (selection, comparability, outcomes) respectively. Each study was considered as low quality (a total score of ≤ 4), moderate quality (a total score between 5–6), or high quality (a total score of ≥ 7).
