# Supplementary material for: Shock index and shock index, pediatric age-adjusted as predictors of mortality in pediatric patients with trauma: A systematic review and meta-analysis
Source: PLoS One. 2024 Jul 18;19(7):e0307367. doi: 10.1371/journal.pone.0307367 (PMC11257222; doi:10.1371/journal.pone.0307367)
Supplement: S3 Table — (DOCX) [file pone.0307367.s004.docx]

**S3 Table. Additional characteristics of each included study**

| **Study ID** | **Study center(s)** | **Exclusion** | **Severity of the included patients** | **Age** | **Time of assessment** |
| --- | --- | --- | --- | --- | --- |
| **2015 Acker** | Children's Hospital Colorado and Denver Health Medical Center | N/A | ISS >15 | 4–16 y | At ED presentation |
| **2017 Linnaus** | Level 1 Pediatric trauma centers | Age <4 or ≥17 years, patients with an unknown ISS or an ISS ≤15 | ISS >15 | 4–16 y | In the ED |
| **2018 Vandewalle** | Level-one pediatric trauma center (Riley Hospital for Children at Indian University Health) | Age <4 or >16 years; if they presented to the hospital more than 12 h after injury | ISS of ≥15 | 4–16 y | At the time of arrival in the hospital |
| **2019 Nordin** | Either ACS Verified or state designated as a Level I or II trauma centers (participants in TQIP-P) | Patients who died prior to ED arrival and those with an ISS<15; patients who arrived in the ED>120 min after EMS arrival; inter-hospital transfers; any records with incomplete data | ISS ≥ 15 & AIS ≥2 | 1–15 y | At the trauma scene and on ED arrival. |
| **2019 Traynor - a** | Pietermaritzburg Metropolitan Trauma Service | Children aged <1 year; patients who did not undergo resuscitation or were deemed dead upon arrival; those patients missing ISS, HR or BP measurement | ISS ≥ 25, 5.4% | 1–17 y | At the time of initial assessment in-hospital |
| **2019 Traynor - b** | Mayo Clinic | Children aged <1 year; patients who did not undergo resuscitation or were deemed dead upon arrival; those patients missing ISS, HR, or BP measurement | ISS ≥ 25, 6.7% | 1–17 y | At the time of initial assessment in-hospital |
| **2020 Marenco** | Military treatment facilities in US and around the world (data from the DODTR) | Patients without vital signs recorded upon arrival to the initial level of care; those patients whose only records were from tertiary care centers (defined as a Role IV center or above) | ISS (mean), 11.8 ± 9.6;  ISS > 15, 31.8% | <18 y | Upon arrival to the initial level of care |
| **2021 Austin** | Trauma centers across the  US (data from the NTDB) | Patients who were not treated at a trauma center; had no data on transfers; had no documented ED, HR, or SBP | N/A | 1–14 y | At the ED presentation |
| **2021 Marenco** | Role II or III combat surgical hospital (data from the DODTR) | All those patients who lacked a recorded HR or SBP in the field and upon arrival to the initial level of care; those patients whose first record of care was at a tertiary care facility (which we defined as a Role IV center and above) | mean ISS (SD), 12 (10); ISS>15, 30.5% | ≤17 y | Both pre-hospital and upon arrival to the initial level of care with surgical capabilities |
| **2022 Georgette** | Trauma centers across the US (data from the TQP-PUF/NTDB) | Patients aged <12 months; burn-dominant injuries; children lacking signs of life on arrival; or who had incompatible (HR divided by SBP of <0.2 or >5) or missing values for vital signs | ISS >25, 3.1% | 1–16 y | First available vital signs from the ED |
| **2022 Raythatha** | Level 1 Australian Metropolitan Pediatric trauma center | Patients with burns, transfers from other hospitals; death before arrival to the hospital | Median ISS (IQR), 9 (2-21);  ISS > 24, 22.0%; | 0–16 y | First available vital observations made by EMS (pre-arrival observations); first available observations within 30 min of arrival at the trauma center (arrival observations) |
| **2022 Stevens** | American College of Surgeons verified and regional Level I pediatric trauma center (Children’s Hospital Colorado) | N/A | ISS, mean (SD):18.7 (13.1) | ≤18 y | Initial pre-hospital vital signs (vital signs at the scene of injury); first set of vital signs upon arrival to the ED trauma bay |

AIS = Abbreviated Injury Scale, DODTR = Department of Defense Trauma Registry, EMS = emergency medical services, ED = emergency department, HR = heart rate, IQR = interquartile range, ISS = Injury Severity Score, N/A = not available, NTDB = National Trauma Data Bank, SD = standard deviation, SBP = systolic blood pressure, TQIP-P = Pediatric Trauma Quality Improvement Program database, TQP-PUF = Trauma Quality Program Participant Use File, US = United States.
