# Supplementary material for: Shock index and shock index, pediatric age-adjusted as predictors of mortality in pediatric patients with trauma: A systematic review and meta-analysis
Source: PLoS One. 2024 Jul 18;19(7):e0307367. doi: 10.1371/journal.pone.0307367 (PMC11257222; doi:10.1371/journal.pone.0307367)
Supplement: S4 Table — (DOCX) [file pone.0307367.s005.docx]

**S4 Table. Normal pediatric vital signs based on age with calculated SIPA cutoff values**

| **Age** | **Heart rate, beats/min** | **Systolic blood pressure, mmHg** | **Diastolic blood pressure, mmHg** | **Respiratory rate, breaths/min** | **SIPA cutoff value** |
| --- | --- | --- | --- | --- | --- |
| **2015 Acker; 2017 Linnaus; 2018 Vandewalle** | | | | | |
| 4-6 years | 65-110 | 90-110 | 60-75 | 20-25 | 1.22 |
| 7-12 years | 60-100 | 100-120 | 60-75 | 14-22 | 1.0 |
| ≥13 years | 55-90 | 100-135 | 65-85 | 12-20 | 0.9 |
| **2019 Nordin; 2021 Austin** | | | | | |
| 1-3 years | 70-110 | 90-110 | N/A | N/A | 1.2 |
| 4-6 years | 65-110 | 90-110 | N/A | N/A | 1.2 |
| 7-12 years | 60-100 | 100-120 | N/A | N/A | 1.0 |
| ≥13 years | 55-90 | 100-135 | N/A | N/A | 0.9 |
| **2019 Traynor – a; 2019 Traynor – b** | | | | | |
| 1-3 years | 70-110 | 90-110 | N/A | N/A | 1.22 |
| 4-6 years | 65-110 | 90-110 | N/A | N/A | 1.22 |
| 7-12 years | 60-100 | 100-120 | N/A | N/A | 1.0 |
| ≥13 years | 55-90 | 100-135 | N/A | N/A | 0.9 |
| **2020 Marenco; 2021 Marenco** | | | | | |
| 0-3 years | 70-110 | 90-110 | N/A | N/A | 1.2 |
| 4-6 years | 65-110 | 90-110 | N/A | N/A | 1.2 |
| 7-12 years | 60-100 | 100-120 | N/A | N/A | 0.9 |
| 13-17 years | 55-95 | 100-135 | N/A | N/A | 0.9 |
| **2022 Georgette** | | | | | |
| 1-3 years | 110 | 90 | N/A | N/A | 1.2 |
| 4-6 years | 110 | 90 | N/A | N/A | 1.2 |
| 7-12 years | 100 | 100 | N/A | N/A | 1.0 |
| ≥13 years | 90 | 100 | N/A | N/A | 0.9 |
| **2022 Raythatha** | | | | | |
| Less than 1 | N/A | N/A | N/A | N/A | 2.0 |
| 1-3 years | N/A | N/A | N/A | N/A | 1.5 |
| 4-6 years | N/A | N/A | N/A | N/A | 1.25 |
| 7-12 years | N/A | N/A | N/A | N/A | 1.0 |
| ≥13 years | N/A | N/A | N/A | N/A | >0 |
| **2022 Stevens (at the trauma scene)** | | | | | |
| Less than 1 | N/A | N/A | N/A | N/A | 1.65 |
| 1-6 years | N/A | N/A | N/A | N/A | 1.52 |
| 7-12 years | N/A | N/A | N/A | N/A | 1.26 |
| ≥13 years | N/A | N/A | N/A | N/A | 1.60 |
| **2022 Stevens (at the hospital)** | | | | | |
| Less than 1 | N/A | N/A | N/A | N/A | 2.54 |
| 1-6 years | N/A | N/A | N/A | N/A | 1.47 |
| 7-12 years | N/A | N/A | N/A | N/A | 1.31 |
| ≥13 years | N/A | N/A | N/A | N/A | 1.24 |

SIPA = shock index pediatric age-adjusted
