# Supplementary material for: Shock index and shock index, pediatric age-adjusted as predictors of mortality in pediatric patients with trauma: A systematic review and meta-analysis
Source: PLoS One. 2024 Jul 18;19(7):e0307367. doi: 10.1371/journal.pone.0307367 (PMC11257222; doi:10.1371/journal.pone.0307367)
Supplement: S5 Table — (DOCX) [file pone.0307367.s006.docx]

**S5 Table. Subgroup analysis and meta-regression analysis (pre-hospital SIPA)**

| **Variable** |  | **N** | **Sensitivity  (95% CI)** | **Sensitivity  *P*-value^†^** | **Specificity  (95% CI)** | **Specificity  *P*-value^†^** |
| --- | --- | --- | --- | --- | --- | --- |
| Country |  |  |  | 0.8270 |  | 0.002 |
|  | Other countries | 2 | 0.638 (0.487, 0.766) |  | 0.622 (0.541, 0.696) |  |
|  | US | 2 | 0.603 (0.328, 0.825) |  | 0.712 (0.697, 0.726) |  |
| Setting |  |  |  | 0.9598 |  | < 0.001 |
|  | Warzone/combat setting | 1 | 0.615 (0.456, 0.753) |  | 0.584 (0.545, 0.622) |  |
|  | Civilian setting | 3 | 0.625 (0.420, 0.794) |  | 0.706 (0.689, 0.721) |  |
| Type of center |  |  |  | 0.0250 |  | 0.587 |
|  | Single center | 2 | 0.711 (0.594, 0.805) |  | 0.692 (0.644, 0.736) |  |
|  | Multicenter | 2 | 0.539 (0.378, 0.693) |  | 0.652 (0.518, 0.766) |  |
| Data source |  |  |  | 0.0250 |  | 0.587 |
|  | Medical records/trauma registries (non-national) | 2 | 0.711 (0.594, 0.805) |  | 0.692 (0.644, 0.736) |  |
|  | National data registry | 2 | 0.539 (0.378, 0.693) |  | 0.652 (0.518, 0.766) |  |
| Cutoff |  |  |  | 0.0250 |  | 0.587 |
|  | New | 2 | 0.711 (0.594, 0.805) |  | 0.692 (0.644, 0.736) |  |
|  | Typical* | 2 | 0.539 (0.378, 0.693) |  | 0.652 (0.518, 0.766) |  |

CI = confidence interval, SIPA = shock index, pediatric age-adjusted, US = United States.

^*^Typical cutoff values are 1.2 (ages 0–6 years), 1.0 (ages 7–12 years), and 0.9 (ages 13–18 years) for the SIPA

**^†^**In meta-regression analysis, a p-value < 0.05 indicates heterogeneity in sensitivity or specificity, suggesting that the effects vary across the subgroup.
