# Supplementary material for: Shock index and shock index, pediatric age-adjusted as predictors of mortality in pediatric patients with trauma: A systematic review and meta-analysis
Source: PLoS One. 2024 Jul 18;19(7):e0307367. doi: 10.1371/journal.pone.0307367 (PMC11257222; doi:10.1371/journal.pone.0307367)
Supplement: S6 Table — (DOCX) [file pone.0307367.s007.docx]

**S6 Table. Subgroup analysis and meta-regression analysis (SIPA at the hospital)**

| **Variable** |  | **N** | **Sensitivity  (95% CI)** | **Sensitivity  *P*-value^†^** | **Specificity  (95% CI)** | **Specificity  *P*-value^†^** |
| --- | --- | --- | --- | --- | --- | --- |
| Country |  |  |  | 0.603 |  | 0.772 |
|  | Other countries | 3 | 0.526 (0.369,0.678) |  | 0.698 (0.541,0.820) |  |
|  | US | 6 | 0.632 (0.391,0.821) |  | 0.736 (0.557,0.861) |  |
| Setting |  |  |  | 0.931 |  | 0.432 |
|  | Warzone/Combat setting | 1 | 0.625 (0.545,0.698) |  | 0.580 (0.559,0.602) |  |
|  | Civilian setting | 8 | 0.603 (0.418,0.762) |  | 0.740 (0.609,0.839) |  |
| Type of center |  |  |  | 0.828 |  | 0.103 |
|  | Single center | 5 | 0.546 (0.463,0.627) |  | 0.792 (0.727,0.845) |  |
|  | Multicenter | 4 | 0.611 (0.358,0.816) |  | 0.621 (0.359,0.827) |  |
| Data source |  |  |  | 0.278 |  | 0.971 |
|  | Medical records/trauma registries (non-national) | 7 | 0.654 (0.502,0.780) |  | 0.726 (0.574,0.840) |  |
|  | National data registry | 2 | 0.490 (0.245,0.739) |  | 0.721 (0.432,0.898) |  |
| Cutoff |  |  |  | 0.501 |  | 0.179 |
|  | New | 2 | 0.461 (0.293,0.638) |  | 0.838 (0.796,0.873) |  |
|  | Typical^*^ | 7 | 0.600 (0.430,0.749) |  | 0.683 (0.531,0.804) |  |

CI = confidence interval, SIPA = shock index, pediatric age-adjusted, US = United States.

^*^Typical cutoff values are 1.2 (ages 0–6 years), 1.0 (ages 7–12 years), and 0.9 (ages 13–18 years) for the SIPA

**^†^**In meta-regression analysis, a p-value < 0.05 indicates heterogeneity in sensitivity or specificity, suggesting that the effects vary across the subgroup.
