# Supplementary material for: Shock index and shock index, pediatric age-adjusted as predictors of mortality in pediatric patients with trauma: A systematic review and meta-analysis
Source: PLoS One. 2024 Jul 18;19(7):e0307367. doi: 10.1371/journal.pone.0307367 (PMC11257222; doi:10.1371/journal.pone.0307367)
Supplement: S7 Table — (DOCX) [file pone.0307367.s008.docx]

**S7 Table. Subgroup analysis and meta-regression analysis (SI)**

| **Variable** |  | **N** | **Sensitivity  (95% CI)** | **Sensitivity  *P*-value^†^** | **Specificity  (95% CI)** | **Specificity  *P*-value^†^** |
| --- | --- | --- | --- | --- | --- | --- |
| Country |  |  |  | 0.9079 |  | 0.4611 |
|  | Other countries | 2 | 0.756 (0.499,0.906) |  | 0.360 (0.161,0.623) |  |
|  | US | 5 | 0.747 (0.490,0.900) |  | 0.483 (0.311,0.658) |  |
| Setting |  |  |  | 0.4949 |  | 0.1832 |
|  | Warzone/Combat setting | 1 | 0.842 (0.775,0.892) |  | 0.246 (0.227,0.265) |  |
|  | Civilian setting | 6 | 0.718 (0.531,0.851) |  | 0.485 (0.342,0.630) |  |
| Type of center |  |  |  | 0.6119 |  | 0.3421 |
|  | Single center | 2 | 0.680 (0.474,0.833) |  | 0.559 (0.431,0.680) |  |
|  | Multicenter | 5 | 0.743 (0.580,0.858) |  | 0.402 (0.239,0.591) |  |
| Data source |  |  |  | 0.3736 |  | 0.9274 |
|  | Medical records/trauma registries (non-national) | 4 | 0.811 (0.584,0.929) |  | 0.441 (0.248,0.654) |  |
|  | National data registry | 3 | 0.690 (0.489,0.839) |  | 0.456 (0.248,0.680) |  |
| Cutoff |  |  |  | 0.4949 |  | 0.1832 |
|  | New | 1 | 0.842 (0.775,0.892) |  | 0.246 (0.227,0.265) |  |
|  | Typical^*^ | 6 | 0.718 (0.531,0.851) |  | 0.485 (0.342,0.630) |  |
| Time |  |  |  | 0.3281 |  | 0.6067 |
|  | At the trauma scene (pre-hospital) | 1 | 0.559 (0.485,0.630) |  | 0.546 (0.527,0.565) |  |
|  | At the hospital | 6 | 0.732 (0.605,0.829) |  | 0.430 (0.278,0.597) |  |

CI = confidence interval, SI = shock index, US = United States.

^*^Typical cutoff value is 0.9 for the SI.

^†^In meta-regression analysis, a p-value < 0.05 indicates heterogeneity in sensitivity or specificity, suggesting that the effects vary across the subgroup.
