# Supplementary figures and images for: Shock index and shock index, pediatric age-adjusted as predictors of mortality in pediatric patients with trauma: A systematic review and meta-analysis
Source: PLoS One. 2024 Jul 18;19(7):e0307367. doi: 10.1371/journal.pone.0307367 (PMC11257222; doi:10.1371/journal.pone.0307367)

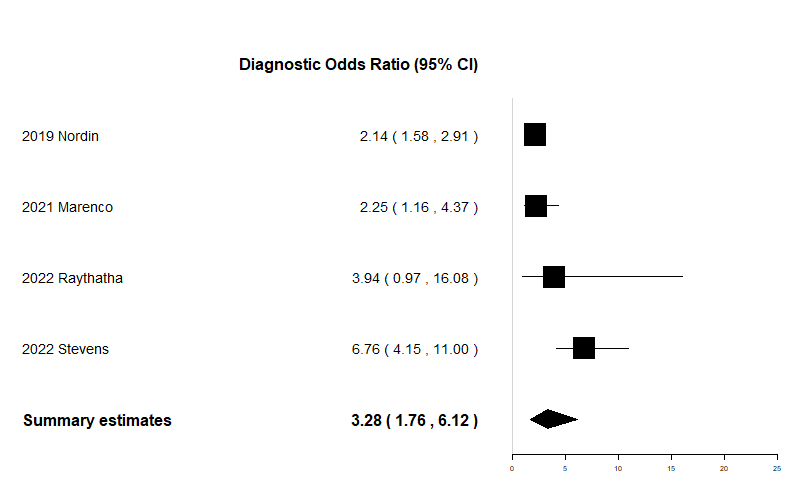

Supplement: S1 Fig — CI = confidence interval, SIPA = shock index, pediatric age-adjusted. (TIF) [file pone.0307367.s009.tif]

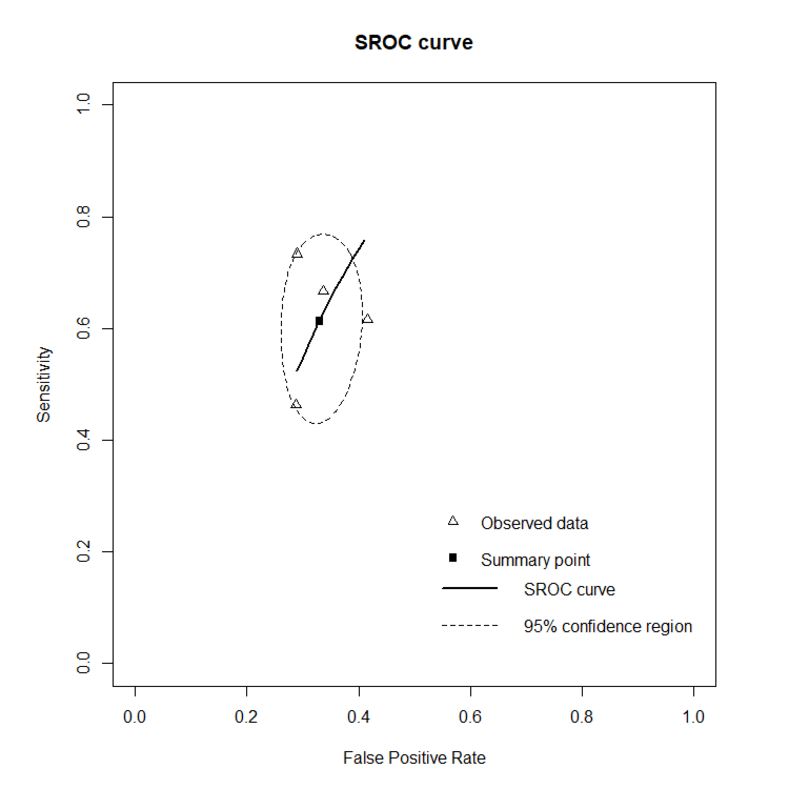

Supplement: S2 Fig — The area under the curve of the SROC was 0.689. SIPA = shock index, pediatric age-adjusted. (TIF) [file pone.0307367.s010.tif]

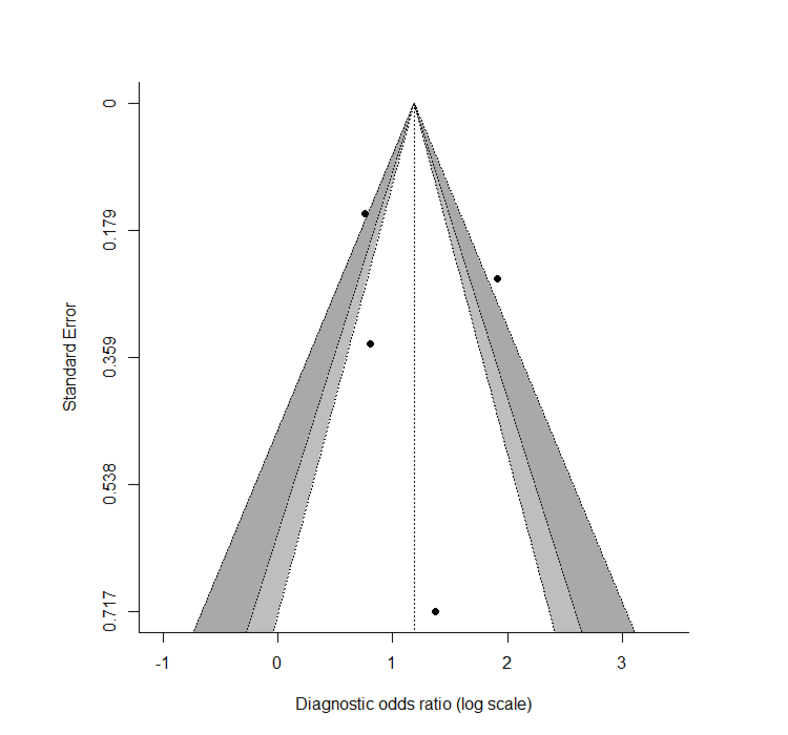

Supplement: S3 Fig — There was no significant publication bias observed by Egger’s test (p = 0.8066). SIPA = shock index, pediatric age-adjusted. (TIF) [file pone.0307367.s011.tif]

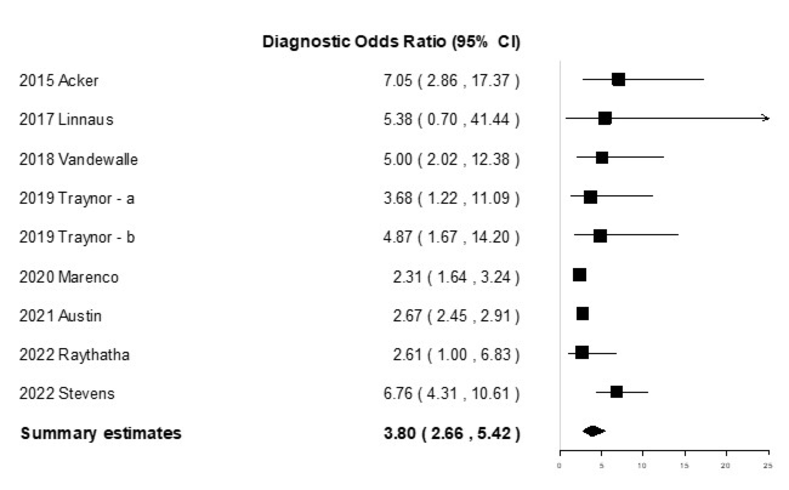

Supplement: S4 Fig — CI = confidence interval, SIPA = shock index, pediatric age-adjusted. (TIF) [file pone.0307367.s012.tif]

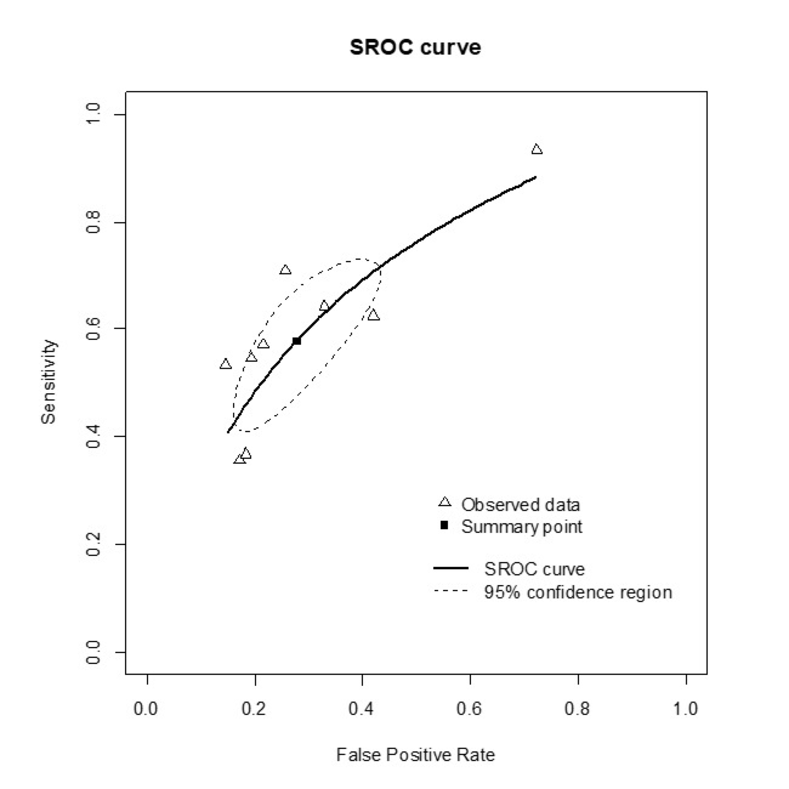

Supplement: S5 Fig — The area under the curve of the SROC was 0.693. SIPA = shock index, pediatric age-adjusted. (TIF) [file pone.0307367.s013.tif]

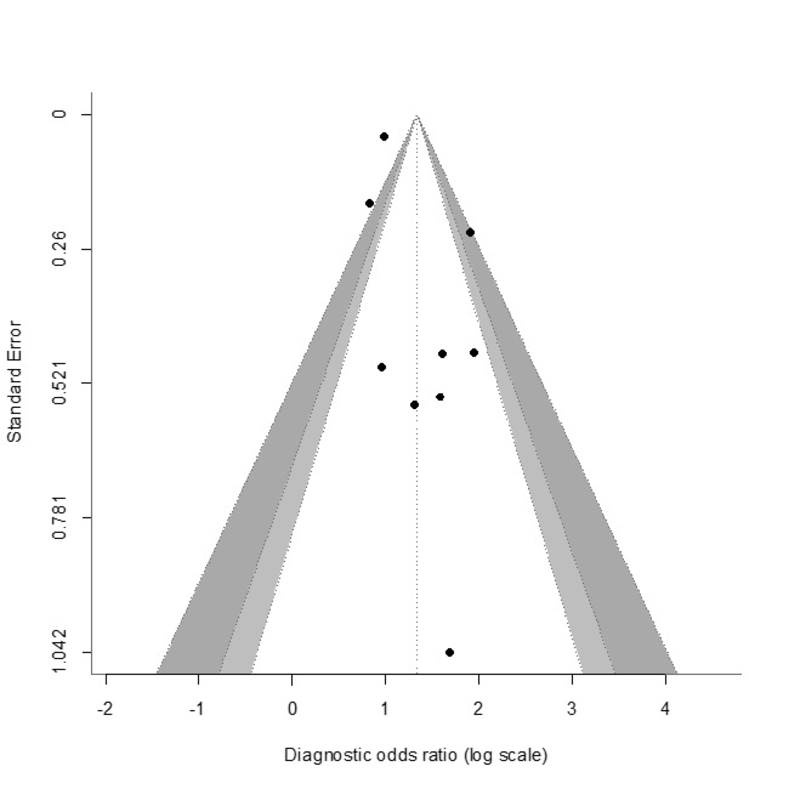

Supplement: S6 Fig — There was no significant publication bias observed by Egger’s test (p = 0.256). SIPA = shock index, pediatric age-adjusted. (TIF) [file pone.0307367.s014.tif]

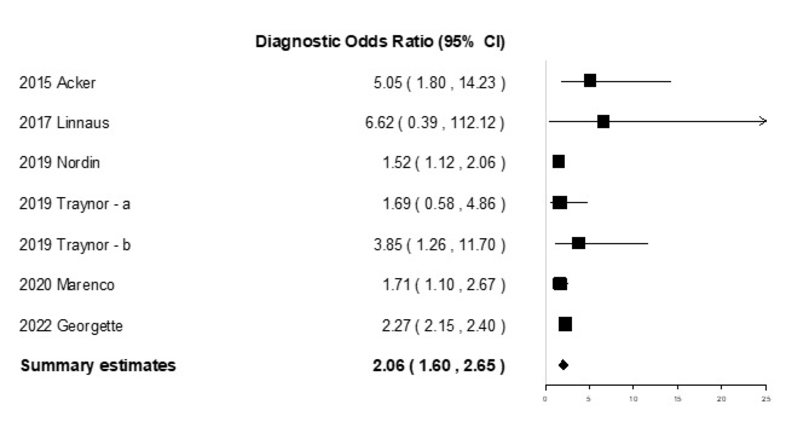

Supplement: S7 Fig — CI = confidence interval, SI = shock index. (TIF) [file pone.0307367.s015.tif]

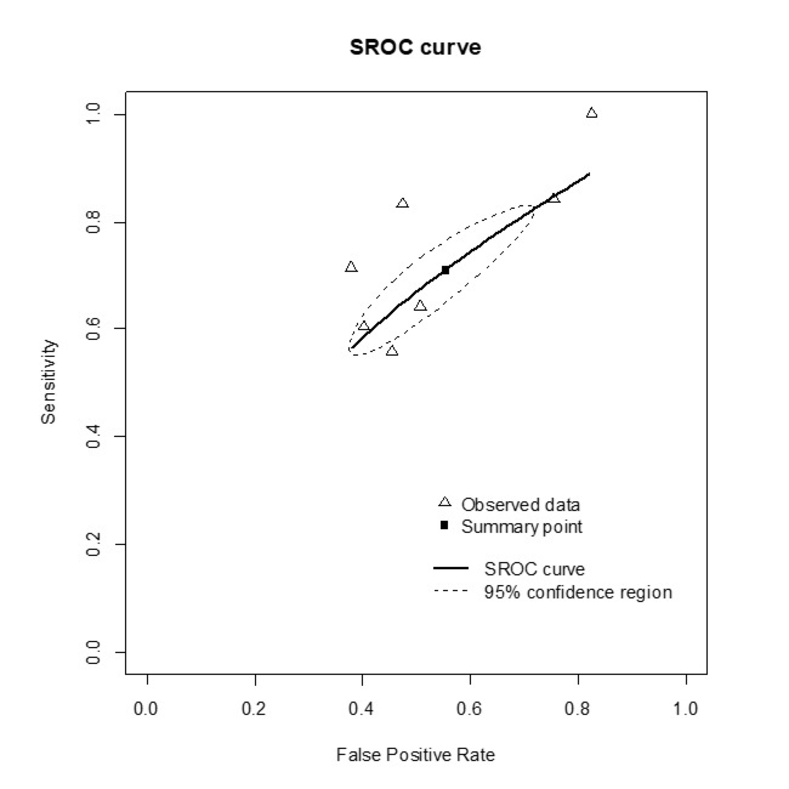

Supplement: S8 Fig — The area under the curve of the SROC was 0.618. SI = shock index. (TIF) [file pone.0307367.s016.tif]

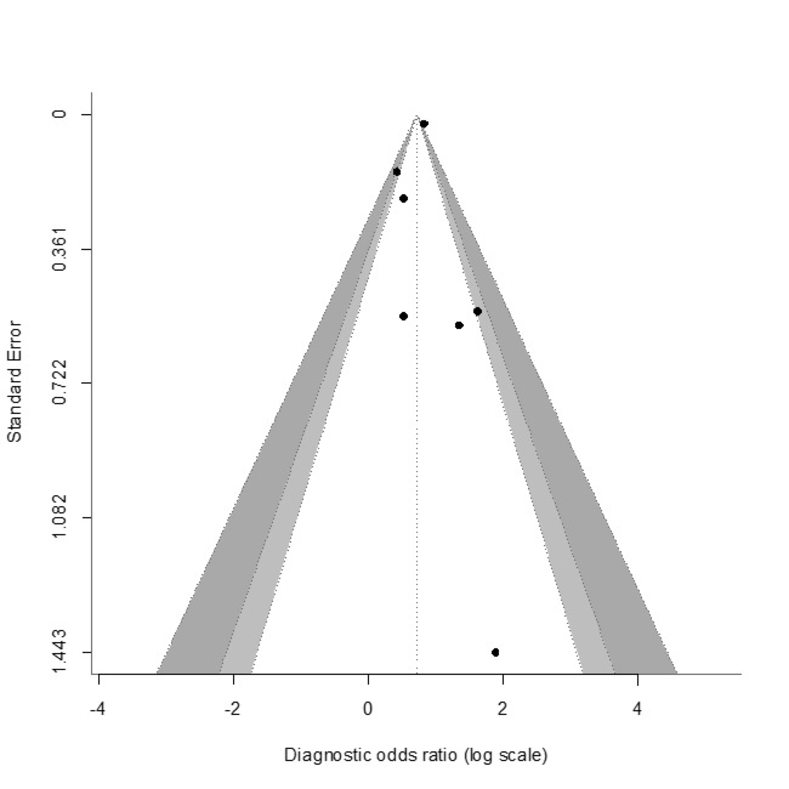

Supplement: S9 Fig — There was no significant publication bias observed by Egger’s test (p = 0.223). SI = shock index. (TIF) [file pone.0307367.s017.tif]
